# Resveratrol Derivative Exhibits Marked Antiproliferative Actions, Affecting Stemness in Pancreatic Cancer Cells

**DOI:** 10.3390/ijms24031977

**Published:** 2023-01-19

**Authors:** Rosalba Florio, Barbara De Filippis, Serena Veschi, Viviana di Giacomo, Paola Lanuti, Giulia Catitti, Davide Brocco, Annalisa di Rienzo, Amelia Cataldi, Ivana Cacciatore, Rosa Amoroso, Alessandro Cama, Laura De Lellis

**Affiliations:** 1 Department of Pharmacy, University “G. D’Annunzio” Chieti-Pescara, 66100 Chieti, Italy; 2 Department of Medicine and Aging Sciences, University “G. D’Annunzio” Chieti-Pescara, 66100 Chieti, Italy; 3 Center for Advanced Studies and Technology (C.A.S.T.), University “G. D’Annunzio” Chieti-Pescara, 66100 Chieti, Italy

**Keywords:** pancreatic ductal adenocarcinoma, polyphenols, natural compounds, phytoalexin, CD133, EpCAM

## Abstract

Pancreatic cancer (PC) is one of the deadliest malignancies, with an increasing incidence and limited response to current therapeutic options. Therefore, more effective and low-toxic agents are needed to improve PC patients’ outcomes. Resveratrol (RSV) is a natural polyphenol with multiple biological properties, including anticancer effects. In this study, we explored the antiproliferative activities of newly synthetized RSV analogues in a panel of PC cell lines and evaluated the physicochemical properties of the most active compound. This derivative exhibited marked antiproliferative effects in PC cells through mechanisms involving DNA damage, apoptosis induction, and interference in cell cycle progression, as assessed using flow cytometry and immunoblot analysis of cell cycle proteins, PARP cleavage, and H2AX phosphorylation. Notably, the compound induced a consistent reduction in the PC cell subpopulation with a CD133^+^EpCAM^+^ stem-like phenotype, paralleled by dramatic effects on cell clonogenicity. Moreover, the RSV derivative had negligible toxicity against normal HFF-1 cells and, thus, good selectivity index values toward PC cell lines. Remarkably, its higher lipophilicity and stability in human plasma, as compared to RSV, might ensure a better permeation along the gastrointestinal tract. Our results provide insights into the mechanisms of action contributing to the antiproliferative activity of a synthetic RSV analogue, supporting its potential value in the search for effective and safe agents in PC treatment.

## 1. Introduction

Pancreatic cancer (PC) is currently the third most common cause of cancer-related mortality worldwide, with a 5-year survival rate of approximately 9% [1,2,3]. Its poor prognosis is primarily due to late diagnosis, high risk of metastatic spread, limited response to radio- and chemotherapy, and lack of response to immunotherapy for PC patients [2,4]. At present, standard first-line therapies for unresectable PC patients are represented by combined treatments including gemcitabine/nab-paclitaxel or FOLFIRINOX regimen, which improved survival over gemcitabine monotherapy [5]. However, these therapeutic options are rather toxic and limited to PC patients with a good performance status [6]. Thus, more effective and less toxic compounds to improve the long-term survival rate of PC patients are urgently needed. Several strategies are pursued to expand the treatment options in PC, including new drug discovery, repurposing of non-anticancer drugs, and the use of natural compounds and their derivatives exhibiting antitumor properties [7,8,9,10,11,12,13,14].

**Figure 1:**
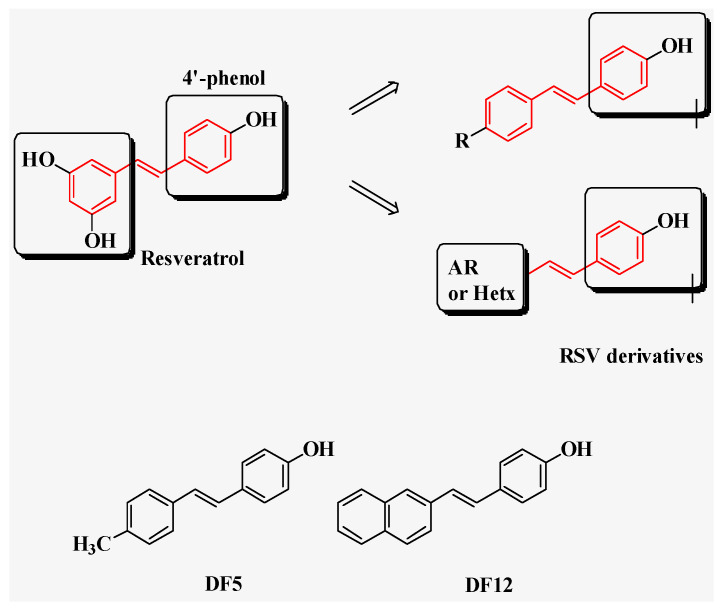
Chemical structures of RSV and its derivatives DF5 and DF12.

Natural polyphenols from vegetables and fruits are potent sources of bioactive, safe, and cost-effective compounds that are able to modulate several cell signaling pathways [15,16]. In this regard, resveratrol (3,5,4′-trihydroxy-trans-stilbene, RSV, Figure 1) is a stilbenoid polyphenol (phytoalexin) that was first isolated from *Veratrum grandiflorum O. Loes* and found also in more than 70 plant species. RSV represents one of the most important natural compounds displaying pleiotropic activities in cell biology, including anti-diabetic, anti-hyperlipidemic, anti-inflammatory, immuno-modulatory, anti-viral, cardio-protective, neuroprotective, chemopreventive, and anti-cancer properties [17]. In particular, RSV has been reported to exert anticancer activities, as single agent or in combination, in an increasing number of cell and animal cancer models by modulating a plethora of pivotal cancer-related pathways [18,19,20,21,22,23,24,25]. Unfortunately, RSV has both poor bioavailability and rapid metabolism, which possibly limit the in vivo translation of encouraging in vitro results [16,26]. Recently, different medicinal chemistry-based approaches were developed to improve the pharmacokinetic properties of RSV, since the stilbene scaffold represents a suitable tool for chemical modifications [27,28,29,30]. In this regard, we previously synthesized a large series of simplified RSV analogues in which we retained the 4′-phenol moiety, which appears crucial for antioxidant and antiproliferative properties [31,32], whereas the 3,5-dihydroxy moiety was replaced with *para*- or *ortho*-substituted aromatic rings, or with different heterocycles. Such RSV derivatives showed relevant biological activities, including antiproliferative properties [33,34,35,36]. Among others, compound 5 and compound 12 (DF5 and DF12, respectively, in Figure 1) appeared as the most active RSV derivatives in inhibiting cell viability across three distinct PC cell lines, as compared to the parent compound RSV [35].

In the present study, to gain insights into the unexplored potential of the newly synthetized RSV derivatives as antiproliferative agents, we investigated their ability in modulating cancer-relevant molecular pathways in a panel of PC cell lines with distinct genetic profiles. DF5 emerged as the most effective RSV derivative in inhibiting PC cell viability and proliferation through mechanisms involving interference in cell cycle progression. It is worth noting that the effects on the induction of both apoptosis and DNA damage were variable among PC cell lines. Notably, DF5 markedly affected the CD133^+^EpCAM^+^ cancer stem-like PC cell subpopulation, associated with drastic effects on cell clonogenicity. Furthermore, its higher stability in human plasma and improved lipophilicity, as compared to RSV, might warrant a better permeation along the gastrointestinal tract, thus supporting the value of the RSV derivative DF5 in the search for novel and effective compounds for PC treatment.

## 2. Results

### 2.1. Effects of RSV and Its Analogues on the Viability of Three Distinct PC Cell Lines

**Figure 2:**
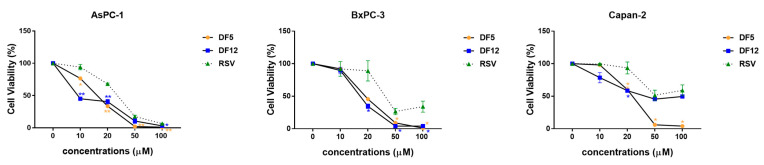
RSV and its derivatives DF5 and DF12 affect cell viability in PC cell lines. Cell viability was assessed using MTT assays after incubation for 72 h with RSV, DF5, or DF12 at the indicated concentrations. Data shown are means ± SD of two independent experiments with quintuplicate determinations. * Statistically significant differences as compared to vehicle (0 µM) (* *p* < 0.05; ** *p* < 0.01).

The effects of resveratrol (RSV) and of two of its analogues (Figure 1) on the viability of three different pancreatic cancer (PC) cell lines, namely AsPC-1, BxPC-3, and Capan-2, were analyzed using MTT (Figure 2). Overall, the compounds inhibited PC cell viability in a dose-dependent manner, although with distinct sensitivities across the three PC cell lines. (Figure 2 and Table 1). In particular, DF5 showed consistent and relevant antiproliferative effects across the three PC cell lines (Figure 2 and Table 1). DF12 had IC_50_ values comparable to those obtained with DF5 in AsPC-1 and BxPC-3 cells, whereas it displayed a higher IC_50_ value in the Capan-2 cell line (Figure 2 and Table 1). Conversely, RSV showed the highest IC_50_ values across the three PC cell lines (Figure 2 and Table 1).

### 2.2. Effects of RSV and Its Analogues on Clonogenic Capacity of PC Cell Lines

**Figure 3:**
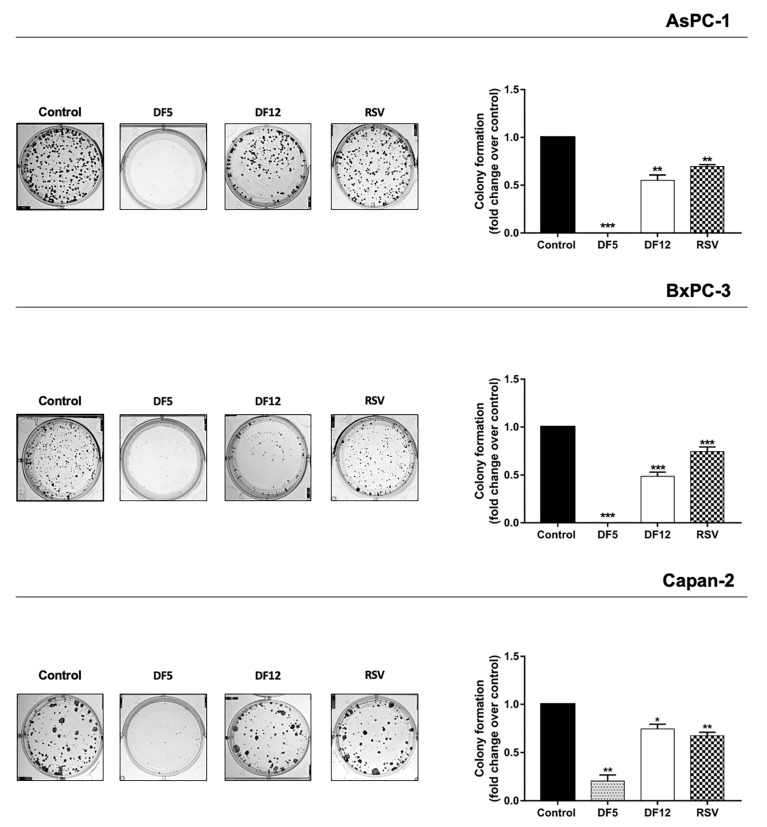
Effect of RSV and its analogues DF5 and DF12 on PC cell clonogenicity. Representative plates of colony formation assays for the three PC cell lines exposed to 20 µM of each compound are shown (**left**). Histograms show the means (±SD) of up to four independent experiments (**right**). Data are expressed as fold change relative to control (* *p* < 0.05; ** *p* < 0.01; *** *p* < 0.001).

We further compared the effects of RSV and of its analogues on the clonogenicity of PC cell lines (Figure 3). Overall, the clonogenicity was reduced after treatment with each compound, as compared to the vehicle control, although with marked differences among the compounds (Figure 3). DF5 showed more drastic, prominent, and consistent effects on PC cell clonogenicity, as compared to DF12 and RSV, and it was able to totally abolish colony formation in AsPC-1 and BxPC-3 cells (Figure 3). DF12 had relevant effects on clonogenic capacity in AsPC-1 and BxPC-3, whereas marginal effects on this activity were observed in Capan-2 cells (Figure 3). RSV significantly, but modestly, affected clonogenic activity across the PC cell lines (Figure 3). Thus, we selected DF5 as a further characterization of its antiproliferative potential.

### 2.3. Effects of RSV and DF5 on Specific Markers Identifying PC Stem Cell Subpopulations

**Figure 4:**
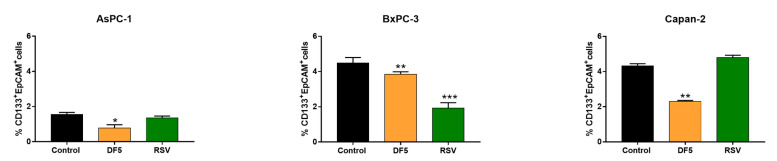
Effects of RSV and DF5 on CD133^+^EpCAM^+^ cancer stem-like PC cell subpopulation. The percentage of the CD133^+^EpCAM^+^ cancer stem-like subpopulation was measured using flow cytometry after incubation for 72 h with DF5, RSV, or with vehicle control. Data shown in the histograms are the mean percentage (± SD) of up to four independent experiments (* *p* < 0.05; ** *p* < 0.01; *** *p* < 0.001).

Clonogenic capacity is a measure of cell self-renewal capacity, which is one of the relevant features of cancer stem cells (CSCs) to maintain proliferating properties [37]. Thus, we investigated using a polychromatic flow cytometry (PFC) method [38] the effects of RSV, or DF5, on CD133 and EpCAM markers, which are typically expressed on the surface of PC stem cells [39,40] (Figure 4). The gating strategy employed for the identification of CD133^+^EpCAM^+^ cells is depicted in Figure S1. In AsPC-1 and Capan-2, the DF5 derivative induced a sharp and statistically significant reduction in the percentages of CD133^+^EpCAM^+^ cancer stem-like PC cell subpopulation, whereas RSV did not affect this subpopulation in the same PC cells (Figure 4). Conversely, in BxPC-3, both treatments had a statistically significant impact on the percentage of the CD133^+^EpCAM^+^ subpopulation and a more marked effect was observed with RSV (Figure 4).

### 2.4. Effects of RSV and DF5 on PC Cell Growth

**Figure 5:**
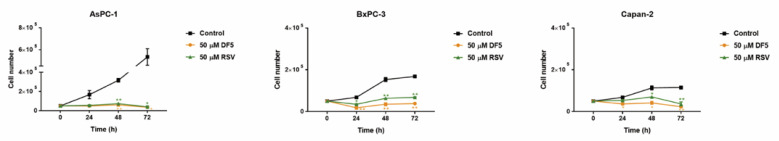
RSV and DF5 affect cell growth in the three PC cell lines. Cell number was assessed using trypan blue exclusion test over a 72 h time course treatment with DF5 (orange curve), RSV (green curve), or with vehicle control (black curve). Data shown are the means (±SD) of three independent experiments (* *p* < 0.05; ** *p* < 0.01).

The impact of RSV, or DF5, on PC cell growth was assessed using a trypan blue exclusion test over a 72 h time course treatment. It is worth noting that the rate of PC cell growth in the absence of treatments was faster in AsPC-1, as compared to BxPC-3 and Capan-2 cell lines (Figure 5). Both compounds significantly, drastically, and comparably affected cell growth in the three PC cell lines at all time points, as compared with the untreated cells (control) (Figure 5).

### 2.5. Effects of RSV and DF5 on the Viability of Normal HFF-1 Cells

**Figure 6:**
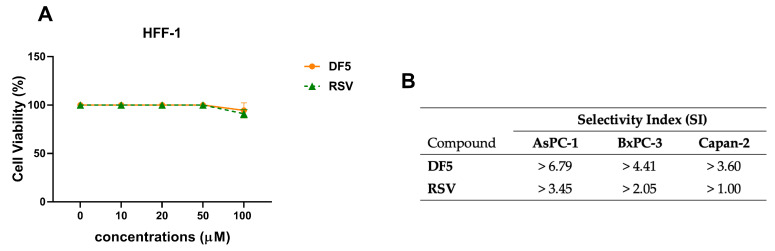
RSV, or DF5, have negligible toxicity against normal HFF-1 fibroblast cells. (**A**) Cell viability was assessed using MTT assay after an incubation of 72 h with RSV (green curve), or DF5 (orange curve), at the indicated concentrations. Data shown are the means ± SD of two independent experiments with quintuplicate determinations. (**B**) Selectivity index (SI) values were calculated for each compound as follows: SI = IC_50_ on normal HFF-1 fibroblast cells/IC_50_ on PC cells. IC_50_ values of RSV and DF5 on HFF-1 cells were both >100 μM. For IC_50_ values of RSV and DF5 in AsPC-1, BxPC-3, and Capan-2 cells see Table 1.

To test the toxicity against normal cells, effects of RSV, or DF5 on normal fibroblast HFF-1 cell viability were assessed using MTT assay. The cells were incubated for 72 h with the two compounds at the indicated concentrations, or with the vehicle (Figure 6A). Both compounds showed negligible toxicity against normal HFF-1 fibroblast cells, displaying IC_50_ values higher than 100 μM in this cell line, which was the highest concentration used in the MTT assays. Notably, DF5 showed up to three-fold higher selectivity index values in the PC cell lines tested, as compared to RSV (Figure 6B).

### 2.6. Effects of RSV and DF5 on Apoptosis in PC Cell Lines

**Figure 7:**
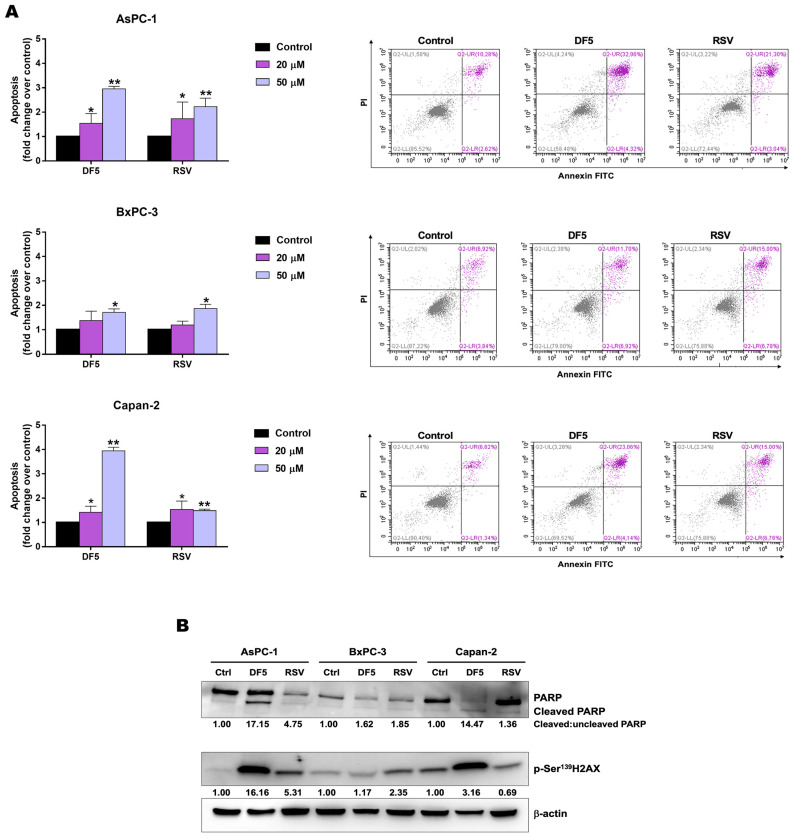
Apoptosis evaluation in PC cell lines treated with DF5, RSV, or with vehicle control. (**A**) Cells were treated for 24 h with DF5, RSV, or with vehicle control at concentrations of 20 μM and 50 μM. Values represented in the histograms (left) are the means ± SD of up to five independent flow cytometry experiments (* *p* < 0.05; ** *p* < 0.01). Dot plots (right) show the results of representative flow cytometry experiments conducted at concentrations of 50 μM. (**B**) Representative Western blots displaying PARP, cleaved PARP, and p-SER^139^H2AX protein expression in PC cell lines treated with 50 μM DF5, RSV, or with vehicle control. Numbers below blots refer to densitometric analyses of immunoreactive bands and represent fold changes in protein expression as compared to controls. For PARP, cleaved:uncleaved PARP ratios are indicated. β−actin was included as a loading control.

To evaluate whether apoptosis could contribute to the decreased PC cell viability and growth observed after RSV, or DF5 treatments, we analyzed annexin-V staining using flow cytometry. AsPC-1, BxPC-3, and Capan-2 cell lines were exposed to a 24 h treatment with RSV, DF5, or with vehicle control, at concentrations of 20 μM and 50 μM. Overall, at a lower concentration (20 μM), both compounds induced a statistically significant apoptosis in AsPC-1 and Capan-2, whereas neither compound affected apoptosis in BxPC-3, as compared to the control (Figure 7A). At a higher concentration (50 μM), the effects of the two compounds on apoptosis appeared more prominent (higher frequencies of Annexin V+ cells) but quite different across the three PC cell lines (Figure 7A). In particular, the DF5 treatment resulted in a significant induction of apoptosis across the three PC cell lines, with a sharper increase in apoptotic/Annexin V+ cells in AsPC-1 and Capan-2, as compared to BxPC-3 (Figure 7A). Regarding RSV, this treatment induced a significant but less marked increment of apoptotic cells across the three PC cell lines (Figure 7A).

Western blot analysis of poly-(ADP-ribose) polymerase (PARP) showed variable effects of the two compounds on PARP cleavage ratios in the three PC cell lines. Specifically, at 50 μM, the DF5 induced a strong increase in cleaved:uncleaved PARP ratios in AsPC-1 and Capan-2, as compared to untreated cells (Figure 7B), with minimal effects on the PARP cleavage ratio in BxPC-3. Differently, RSV affected the PARP cleavage ratio in AsPC-1, but it had small to negligible effects on the PARP cleavage ratios in BxPC-3 and Capan-2, respectively (Figure 7B). H2AX is a histone variant that is phosphorylated at serine 139 as an early stage of DNA damage response [41,42]. To explore the possibility that the observed apoptosis might associate with DNA damage, we analyzed phospho-H2AX (γH2AX) levels after 50 μM RSV, or DF5 treatments of PC cell lines (Figure 7B). Notably, DF5 treatment induced a marked activation of H2AX in AsPC-1, along with less marked or negligible activation of this DNA damage-related marker in Capan-2 and BxPC-3 cells, respectively (Figure 7B). As far as RSV is concerned, this compound increased H2AX phosphorylation in AsPC-1 and to a lesser extent in BxPC-3, whereas no activation of H2AX was observed in Capan-2 (Figure 7B).

Therefore, these findings indicate that apoptosis and DNA damage contribute to the reduced PC cell viability and growth detected after RSV, or DF5 treatments, although with a certain heterogeneity across the three PC cell lines.

### 2.7. Effects of RSV and DF5 on PC Cell Cycle

**Figure 8:**
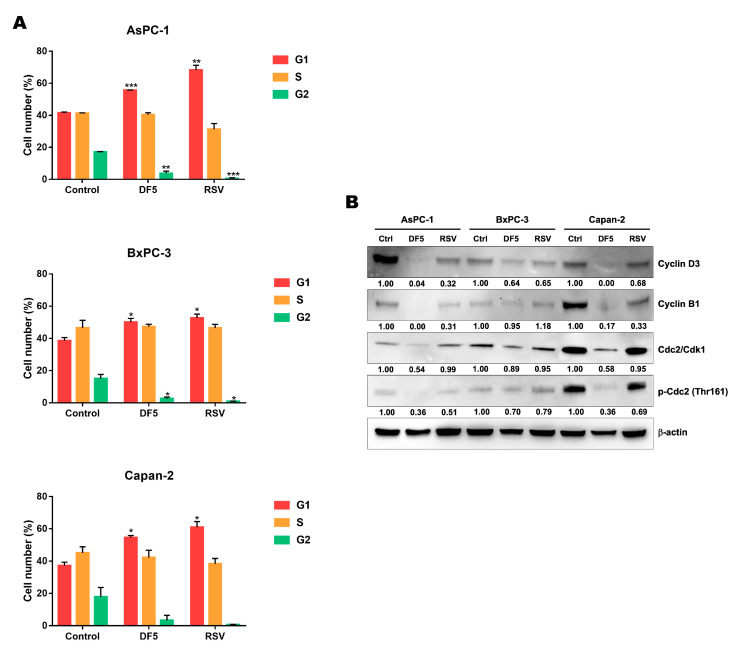
RSV and DF5 affect cell cycle progression in PC cells. (**A**) The histograms show the mean percentage of cells in the different cell cycle phases after 24 h of treatment with 50 μM RSV, DF5, or with vehicle control, as assessed using flow cytometry. Means ± SD are shown (* *p* < 0.05; ** *p* < 0.01; *** *p* < 0.001). (**B**) Representative Western blots showing the expression of Cyclin D3, Cyclin B1, Cdc2/Cdk1, and *p*-Cdc2 (Thr161) in PC cell lines treated with 50 μM DF5, RSV, or with vehicle control. Numbers below blots refer to densitometric analyses of immunoreactive bands and represent fold changes in protein expression as compared to controls. β−actin was included as a loading control.

To explore whether the decreased PC cell viability and proliferation observed after RSV or DF5 treatments could be associated with cell cycle perturbation, we analyzed the effects of the two compounds on PC cell cycle distribution. Overall, the flow cytometry analyses showed that both treatments profoundly altered the cell cycle, with similar patterns in the three PC cell lines (Figure 8A). In particular, 50 μM RSV or DF5 promoted a statistically significant accumulation of cells at the G0/G1 phase across the three PC cell lines after a 24 h treatment, along with a drastic depletion of cells in the G2/M phase, as compared with untreated cells; however, in Capan-2 it did not reach statistical significance (Figure 8A).

The immunoblot analyses showed alterations in cell cycle regulatory protein expression promoted by both compounds in the three PC cell lines, which were in line with the flow cytometry results. Generally, it should be noted that more marked effects in protein levels were observed after DF5 treatment, as compared with RSV treatment at the same concentration in the PC cell lines (Figure 8B). Specifically, DF5 induced a clear-cut reduction or even an abolishment in the expression of cyclin D3, whereas the reduction in cyclin D3 expression was present but less marked with RSV treatment (Figure 8B). In addition, DF5 and, to a lesser extent, RSV caused a reduction in the expression of the cyclin B1/CDK1 complex in AsPC-1 and Capan-2 cell lines, also through Cdc2/CDK1 dephosphorylation at Thr-161. Conversely, these effects were much less evident in BxPC-3 (Figure 8B).

Together, these findings suggest that the reduction in PC cell viability and proliferation observed after RSV or DF5 treatments are at least in part associated with cell cycle perturbation.

### 2.8. Evaluation of DF5 Physicochemical Properties and Chemical Stability in Assay Conditions

Drug-like properties were studied to evaluate the adsorption of DF5 through membranes. As reported in Table 2, DF5 resulted as being insoluble in water and highly lipophilic, more than lead compound RSV (cLogP values 4.67 vs. 2.83, respectively). The high lipophilicity suggests a good propensity of the molecule to distribute in non-aqueous environments, although a limited water solubility may represent a critical parameter for oral adsorption.

The in vitro stability profiles of DF5 are reported in Table 3. DF5 showed a far higher stability in human plasma, as compared to RSV [43], likely due to the absence of labile chemical functions in the presence of plasmatic esterases. Conversely, DF5 exhibited an increased susceptibility in simulated gastric fluid (SGF) and in simulated intestinal fluid (SIF), as assessed by RD% values of 8.87 and 9.36 observed after an incubation time of 1 h in SGF and 3 h in SIF, respectively (Table 3).

A PAMPA-GI model, mimicking the gastrointestinal (GI) tract, was employed to assess the ability of DF5 to cross biological membranes. In agreement with its high lipophilicity, the compound showed a good permeability (P_e_ > 10 nm/s) at each value of the tested pHs (7.4, 6.5, and 5) after 16 h of incubation (Table 4).

HPLC experiments were also performed to evaluate the DF5 chemical stability in assay conditions, specifically over a 72 h incubation in an RPMI 1640 medium enriched with 10% of Fetal Bovine Serum (Figure S2 and Tables S1 and S2). Overall, the chromatographic results showed that only a minimal percentage of DF5 (11%) was degraded after 72 h of incubation, thereby suggesting a good chemical stability for the compound in the assay conditions.

## 3. Discussion

Pancreatic cancer (PC) is one of the deadliest tumors, which presently receives limited benefits from radio-, chemo-, and immunotherapy in terms of survival for patients. Moreover, severe side effects occur in PC patients undergoing systemic chemotherapy, thus there is a need for compounds displaying antitumor properties that spare normal cells. In this regard, natural compounds, including polyphenols, appear as promising, effective, and safe molecules to be exploited as single agents or in combination with standard chemotherapy in the perspective of clinical translation for PC treatment [7,44].

Resveratrol (RSV) received a great attention for its ability to impact on multiple key processes in cancer cell biology, although limitations in terms of low bioavailability and extensive first-pass metabolism hamper its biological activity in vivo [45]. In the present study, we investigated the antiproliferative effects of RSV derivatives in a panel of PC cell lines and explored the physicochemical properties of the most active compound. Specifically, we selected two RSV analogues, namely DF5 and DF12, as the most active compounds in inhibiting PC cell line viability among a large series of previously synthetized stilbene-based derivatives [35]. Remarkably, both derivatives consistently affected PC cell viability in a more marked manner as compared to RSV, displaying IC_50_ values lower than those obtained with the reference compound. This result supports the notion that the replacement of the 3,5-dihydroxy motif of RSV with a *p*-substituted phenyl or with another aromatic ring improves the inhibitory activity of RSV analogues on PC cell viability [35]. It should be also noted that the RSV derivative DF12 had IC_50_ values comparable to those acquired with DF5 in AsPC-1 and BxPC-3 cells, whereas it displayed a higher IC_50_ value in the Capan-2 cell line. It is likely that these differences in sensitivity reflect in part differences in the structure of single substituents and in part differences in the genetic backgrounds of the three tested PC cell lines. It is worth noting that Capan-2 tended to be less sensitive to all compounds, but at the maximum tested concentration (100 µM) DF5 was the most effective in achieving an almost complete loss of cell viability also in this cell line, drastically lowering the residual viability. Conversely, the decrease in Capan-2 cell viability after DF12 or RSV treatments reached a plateau already at concentrations of 50 µM, with a residual viability higher than 50%. A drastic lowering of the residual viability is desirable in the search of agents owing antiproliferative properties to be exploited in the perspective of clinical translation. In line with these results, clonogenic assays also showed that DF5 has stronger effects in reducing or even in abolishing colony formation across all the tested PC cell lines, as compared to DF12 and RSV. It should be noted that DF5 drastically affects the clonogenic capacity of PC cells, even at concentrations that do not achieve dramatic effects in other assays. This greater sensitivity of clonogenic assay is consistent with what we observed in previous studies with different compounds [10,11]. It is well-known that clonogenic capacity is a measure of self-renewal ability of cancer cells, which in turn represents a hallmark of the cell subpopulation having stem-like properties [37]. In this regard, we explored the impact of DF5 treatment on the expression of cell surface antigens that have been recognized to mark stem cell-like properties in PC cells [39,46]. Consistent with the clonogenic assay results, DF5 decreased the CD133^+^EpCAM^+^ cancer stem-like cell subpopulation across all the PC cell lines. It should be noted that RSV treatment in BxPC-3 induced a more marked reduction in the stem cell subpopulation identified by CD133 and EpCAM cell surface markers, as compared to DF5. This result would not appear in line with that obtained in clonogenic assays performed in the same PC cell line. The reason of this discrepancy is unclear, but one possible explanation is that in BxPC-3 cells DF5 drastically decreases an enlarged population of cancer cells having stem-like properties, but only in part recognized by the selected surface markers, whereas RSV preferentially affects the stem-like population of BxPC-3 cells enriched for CD133 and EpCAM cancer stem cell markers. Notably, DF5 showed good selectivity index (SI) values toward PC cell lines, as compared to the normal HFF-1 cell line, with SI values for DF5 even greater than those obtained with the lead compound RSV across the three PC cell lines. Albeit encouraging, this is only a first-line evidence of the safety profile of DF5. Further studies will be necessary to clarify the actual toxicity of the compound in vivo. As far as cell growth is concerned, both treatments drastically reduced the cell proliferation across the three PC cell lines. Notably, it has been previously reported that RSV affects growth and viability in several cancer cell models at least in part by altering cell cycle phase distribution, along with the induction of apoptosis [21,47,48]. Interestingly, these effects promoted by RSV were reported to be variable and concentration- and/or time-dependent across distinct human cancer cell lines [24,49,50]. In the present study, we also related the effects of DF5 and RSV on PC cell viability and growth both to apoptosis induction and to interference with PC cell cycle progression. It is worth noting that the contribution of apoptotic cell death to the decreased viability and proliferation observed after treatments was not consistent across the three PC cell lines. Specifically, compounds fostered PC cell apoptosis in a dose-dependent manner, with DF5 showing more evident effects than RSV, especially in AsPC-1 and Capan-2 cancer cells at the highest concentration, as supported by flow cytometry and immunoblot analysis of PARP cleavage. Conversely, in BxPC-3, we observed a certain inconsistency between the results obtained using flow cytometry and Western blot analyses. This discrepancy suggests that, in BxPC-3, apoptotic cell death contributes to a lesser extent to both decreased cell viability and growth observed with DF5, or RSV treatments, and that this might be related to inherent differences in the genetic profiles of the three tested PC cell lines. Considering that RSV has been reported to induce DNA damage in other cancer cells and that DNA damage is a well-known early marker of apoptosis induction [47,51], we explored the possibility that DF5, similar to RSV, elicited this response in our cell experimental setting. Consistent with the results of apoptosis analysis, DF5 activated the DNA damage marker H2AX more effectively than RSV in AsPC-1 and Capan-2 cancer cells, whereas no relevant effects on H2AX activation were observed with both compounds in BxPC-3. Additionally, in this case, the heterogeneity among PC cell lines is likely to be related to their different genetic backgrounds. As far as the cell cycle is concerned, DF5 and RSV drastically and consistently altered cell cycle progression by promoting the accumulation of PC cells in the G0/G1 phase, alongside the near disappearance of cells in the G2/M phase. These results were supported by Western blot analyses, which showed that DF5 affected the expression of cell cycle regulatory proteins even more drastically than RSV. Overall, our results provide evidence that the impairment of PC cell clonogenicity, paralleled by the decrease in CD133^+^EpCAM^+^ cancer stem-like PC cell subpopulation, together with DNA damage, apoptosis, and interference in cell cycle progression all contribute to the antiproliferative effects displayed by the DF5 derivative, with distinct effects in the three PC cell lines.

Regarding drug-like properties, DF5 has both higher stability in human plasma and improved lipophilicity, as compared to RSV, which might ensure a proper interaction with biological membranes and a better permeation along the gastrointestinal (GI) tract. It is worth noting that DF5 was not very stable in the simulated GI fluids. However, considering that the RD% values achieved approximately 10% after 3 h, it is likely that DF5 might be delivered to membranes in adequate percentage to guarantee a good adsorption.

## 4. Materials and Methods

### 4.1. Reagents and Antibodies

The 3-(4,5-Dimethyl-2-thiazolyl)-2,5-diphenyl-2H-tetrazolium bromide (MTT), crystal violet, propidium iodide (PI), RNAse, cell lysis buffer, dimethyl sulfoxide (DMSO), phenylmethylsulfonyl fluoride (PMSF), protease and phosphatase inhibitor cocktails, and trypan blue were purchased from Sigma-Aldrich (St. Louis, MO, USA). The mouse monoclonal anti-PARP antibody, anti-phospho-histone H2A.X (Ser139), mouse monoclonal anti-cdc2 (CDK1) antibody, anti-phospho-cdc2 (Thr161) antibody, and mouse monoclonal anti-cyclin D3 were acquired from Cell Signaling Technology, Inc. (Beverly, MA, USA). The monoclonal anti-β-actin antibody was acquired from Sigma-Aldrich (St. Louis, MO, USA). The monoclonal anti-cyclin B1, goat anti-mouse IgG-horseradish peroxidase (HRP), and goat anti-rabbit IgG-HRP antibodies were purchased from Santa Cruz Biotechnology, Inc. (Dallas, TX, USA).

The phycoerythrin (PE)-conjugated anti-CD133/2 (293C3) antibody was purchased from Miltenyi Biotec (Bologna, Italy). The fluorescein isothiocyanate (FITC)-conjugated anti-CD326 (EpCAM) antibody was purchased from BD (BD, Becton-Dickinson Biosciences, San Jose, CA, USA). The Live/Dead Fixable Far Red Dead cell stain kit (Reactive Dye) was acquired from Thermo Fisher Scientific (Waltham, MA, USA).

### 4.2. Cell Lines and Chemicals

The human pancreatic cancer (PC) cell lines AsPC-1, BxPC-3, Capan-2, and human fibroblast cell (HFF-1) were acquired from American Type Culture Collection (ATCC; Manassas, VA, USA). The AsPC-1 cells are KRAS and p53 mutated, BxPC-3 cells are KRAS wild-type and p53 mutated, and Capan-2 cells are KRAS mutated and p53 wild-type. The cell lines were cultured in an RPMI 1640 medium and supplemented with 10% fetal bovine serum (FBS) at 37 °C, 5% CO_2_.

The resveratrol (RSV) derivatives DF5 and DF12 were synthesized by Dr. De Filippis at the University of G. d’Annunzio Chieti, Italy [35] (chemical structures are presented in Figure 1). RSV was purchased from Sigma-Aldrich (St. Louis, MO, USA). The stock solutions of the different compounds were prepared by dissolving them in DMSO and then they were diluted to the final concentrations in the culture media to perform experiments. In this way, the working solutions were completely clear and devoid of any undissolved material as confirmed by microscopic inspection. The final concentration of the DMSO in the experiments was 0.05% and it showed no cell toxicity.

### 4.3. Cell Viability, IC_50_, and Selectivity Index (SI) Calculation and Cell Growth Assays

The cell viability was assessed using MTT assay, as previously described [52]. Briefly, the cells were seeded in 96-well plates (4 × 10^3^ cells/well) and were treated for 72 h with different concentrations of RSV, DF5, and DF12. The IC_50_ values were extrapolated from dose–response curves and calculated using the CompuSyn software (https://www.combosyn.com/). The selectivity index (SI) values were calculated as ratios between the IC_50_ values of compounds in normal HFF-1 fibroblast cells and the IC_50_ values of the same compounds in the PC cell lines.

The cell counting was performed using the trypan blue exclusion test [52]. Briefly, PC cells were seeded in 24-well plates (5 × 10^4^ cells/well) and then were treated with 50 μM of DF5, RSV, or with vehicle and counted at 24, 48, and 72 h.

### 4.4. Colony Formation Assay

A colony formation assay was performed as previously described [52]. Briefly, the PC cells were seeded in 6-well plates, incubated for 24 h, and then treated for 72 h with 20 μM DF5, DF12, and RSV or with vehicle (DMSO) as indicated. After 4 days, following methanol fixation and crystal violet staining, the colonies of at least 30 cells were counted.

### 4.5. Immunophenotyping

The expression of CD133 and EpCAM surface antigens was evaluated using flow cytometry. Briefly, the PC cells were seeded in 6-well plates and treated for 72 h with 20 μM DF5, RSV, or with vehicle (DMSO). After treatment, the cells were collected in PBS and stained at room temperature in the dark for 30 min with PE-CD133, FITC-EpCAM antibodies, and Reactive Dye. After labeling, the samples were washed in PBS by centrifugation at 1500 rpm for 10 min, to remove excess antibodies. Flow cytometer performance, stability, and data reproducibility were daily checked by using the CS&T quality control Module (BD Biosciences) [53]. The gating strategy and the evaluation of non-specific fluorescence was determined using fluorescence minus one (FMO) controls [54]. The data were acquired with FACSVerse analyzer equipped with a volumetric count module (BD Biosciences, San Jose, CA, USA) and are expressed as the percentage of positive cells.

### 4.6. Cell Cycle Analysis

To perform the cell cycle analysis, the PC cells (3 × 10^5^) were collected, fixed in 70% cold ethanol, and kept at 4 °C overnight. The collected cells were incubated at 4 °C overnight in 5 μg/mL PI (final concentration) and 100 μg/mL RNAse (final concentration). The cell cycle profiles were analyzed as previously described [55].

### 4.7. Apoptosis Assay

The apoptosis assay was performed essentially as previously described [52]. the analyses were performed using a CytoFLEX flow cytometer, using the FL1 detector in linear mode and the CytoExpert software (Beckmann Coulter, Milano, Italy).

### 4.8. Western Blot Analysis

The PC cell lines treated with 50 μM DF5, RSV, or with vehicle (DMSO) were lysed in a cell lysis buffer containing PMSF, protease inhibitors cocktails, and phosphatase inhibitors. The protein lysate quantification and immunoblot analyses were performed as previously described [11,52].

### 4.9. HPLC-UV Assays

The HPLC apparatus was equipped with a Waters 600 HPLC pump (Waters Corporation, Milford, MA, USA) and a Waters 2996 photodiode array detector set at 304 nm. The mobile phase was a mixture of water/acetonitrile/trifluoracetic acid 30:70:0.1% (v/v/v) flushing at 1.0 mL/min. A Thermo Scientific Hypersil ODS (250 × 4.6 mm, 5 µm) column was used for the chromatographic separation. The injection volume was 10 μL.

### 4.10. Solubility and Lipophilicity

The solubility was determined as previously described [56]. The calculated LogP (cLogP) value was determined using ACD LogP software package, version 4.55 (Advanced Chemistry Development Inc., Toronto, ON, Canada).

### 4.11. Enzymatic Stability

The in vitro enzymatic stabilities of DF5 were assessed in human plasma and simulated gastric and intestinal fluids (SGF and SIF, respectively). For the human plasma stability assay, 10 μL of a 10-4 M stock solution of DF5 in acetonitrile was added to a pre-heated (37 °C) plasma fraction and diluted with phosphate buffer (pH 7.4, 0.02 M) to obtain a final volume of 1 mL (80% plasma). The samples (50 µL) were extracted at various times and 200 µL of cold acetonitrile containing 0.5% *v*/*v* of formic acid were employed to stop the enzymatic reaction. After centrifugation for 10 min at 5000 g, the supernatant was removed and analyzed using HPLC [57].

The SGF and SIF, containing pepsin and pancreatin, respectively, were prepared following the USP specifications. The reactions were carried out in a shaking water bath at 37 °C. The drug stock solution (10 μL of a 10^−4^ M) was added to the preheated gastrointestinal fluids and, at fixed time points (0, 15, 30, and 60 min for SGF and 0, 60, 120, and 180 min for SIF), 20 μL of the media were mixed with 200 μL of cold acetonitrile containing 0.5% *v*/*v* of formic acid. The samples were centrifuged for 10 min at 5000× *g* and the supernatants were analyzed using HPLC. The relative difference (RD%) was used to predict the amount of compound decomposed in the presence of gastrointestinal fluids [58].

### 4.12. Parallel Artificial Membrane Permeability Assay (PAMPA)

A PAMPA was used to evaluate the passive intestinal absorption of DF5. The system consisted in a 96-well, MultiScreen-IP PAMPA filter plate (donor plate) equipped with a PVDF membrane filter and a 96-well PTFE acceptor plate. The PVDF membrane was coated with 5 µL of a 2% *w*/*v* lecithin in *n*-dodecane solution. Each well of the donor plate was filled with 150 µL of a buffer solution (pH 7.4) containing 500 µM of DF5, while 300 µL of phosphate buffer were introduced into the acceptor plate. Donor and acceptor solutions, both containing 5% of DMSO *v*/*v*%, were employed to avoid precipitation of the drug. The system was assembled placing the drug-filled donor plate into the acceptor plate and incubated at room temperature for 16 h. Each solution was analyzed using HPLC and the permeability (*P_e_*, nm/s) was determined as previously reported [59].

### 4.13. Statistical Analysis

The data were expressed as the mean ± standard deviation (SD). For cell viability, a statistical analysis was performed using one-way ANOVA followed by a Dunnett’s test for multiple comparisons. For the other analyses, the independent samples Student’s t-test was used to compare the treated to the control samples. *p* values <0.05 were considered statistically significant.

## 5. Conclusions

In conclusion, the RSV derivative DF5 exhibits prominent antiproliferative actions in PC cell lines by affecting key processes in cancer cell biology, including clonogenicity, cell cycle progression, and apoptotic cell death. In particular, this compound affects the subpopulation of PC cells with a CD133^+^EpCAM^+^ stem-like phenotype, indicating its potential in inhibiting the self-renewal capacity of PC cells. Remarkably, DF5 has negligible effects on normal HFF-1 cell viability. Together, our results provide insights into mechanisms of action contributing to the antiproliferative activity of DF5 and support the potential value of the compound in the search for effective and safe agents for PC treatment.

## Figures and Tables

**Table 1:** IC50 values for compounds DF5, DF12, and RSV in PC cell lines.

	IC_50_ (µM)		
Compound	AsPC-1	BxPC-3	Capan-2
DF5	14.72	22.70	27.75
DF12	11.07	18.28	58.05
RSV	29.01	48.73	>100

**Table 2:** Physicochemical properties of RSV and DF5.

	Water Solubility	Classification ^a^	cLogP ^b^
Resveratrol	50 μg/mL [43]	PI	2.83
DF5	<10 μg/mL	PI	4.67

^a^ PI (Practically Insoluble); ^b^ Value is calculated with ACD LogP software package, version 4.55.

**Table 3:** Stability of DF5 in simulated gastric fluid (SGF) at pH 1.2 and in simulated intestinal fluid (SIF) at pH 6.8 with pepsin and pancreatin, respectively, and human plasma.

	Time Point (min)	RD% ^a^
SGF	0	-
	15	4.36
	30	7.10
	60	8.87
SIF	0	-
	60	8.12
	120	9.20
	180	9.36
Human Plasma	stable	

^a^ RD% is relative difference, unstable: RD% > 5%, stable: RD% < 5%.

**Table 4:** PAMPA-GI permeability of DF5.

	pH 7.4 ^a^	pH 6.5 ^a^	pH 5 ^a^
Permeability, *P_e_* (nm s^−1^) ^b^	25.85 (±1.51)	89.97 (±8.85)	60.22 (±2.60)
Classification ^c^	Pe+	Pe+	Pe+

^a^ pH of both donator and acceptor compartment. ^b^ Values are means of three experiments; standard deviation is provided in parentheses. ^c^ Pe+ (indicative of high GI permeation): *P_e_* (nm s^−1^) > 10, Pe+/− (discrete GI permeation): *P_e_* (nm s^−1^) from 1 to 10, Pe- (indicative of low GI permeation): *P_e_* (nm s^−1^) < 1.

## Data Availability

Not applicable.

## Supplementary Materials

The supporting information can be downloaded at: https://www.mdpi.com/article/10.3390/ijms24031977/s1.
