# Supplementary material for: Resveratrol Derivative Exhibits Marked Antiproliferative Actions, Affecting Stemness in Pancreatic Cancer Cells
Source: Int J Mol Sci. 2023 Jan 19;24(3):1977. doi: 10.3390/ijms24031977 (PMC9916441; doi:10.3390/ijms24031977)
Supplement: Supplementary file 1 [file ijms-24-01977-s001.zip › ijms-2134278-supplementary.pdf]

Supplementary Information

Resveratrol Derivative Exhibits Marked Antiproliferative Actions, Affecting Stemness in Pancreatic Cancer Cells

Rosalba Florio [1], Barbara De Filippis [1], Serena Veschi [1], Viviana di Giacomo [1], Paola Lanuti [2,3], Giulia Catitti [2,3], Davide Brocco [1], Annalisa di Rienzo [1], Amelia Cataldi [1], Ivana Cacciatore [1], Rosa Amoroso [1], Alessandro Cama [1,*] and Laura De Lellis [1,*]

[1] Department of Pharmacy, University "G. D'Annunzio" Chieti-Pescara, 66100 Chieti, Italy;

[2] Department of Medicine and Aging Sciences, University "G. D'Annunzio" Chieti-Pescara, 66100 Chieti, Italy

[3] Center for Advanced Studies and Technology (C.A.S.T.), University "G. D'Annunzio" Chieti-Pescara, 66100 Chieti, Italy

[*]Correspondence: alessandro.cama@unich.it; laura.delellis@unich.it

Figure S1. Gating strategy for the identification of CD133$^+$EPCAM$^+$ cells. Cells were identified on a FSC-A/SSC-A dot-plot, then singlets and viable cells were gated, to finally study the CD133$^+$EpCAM$^+$ compartment (**A**). The gates were placed on the basis of the fluorescence minus one control (FMO), both for CD133 (**B**) and EpCAM (**C**) stainings.

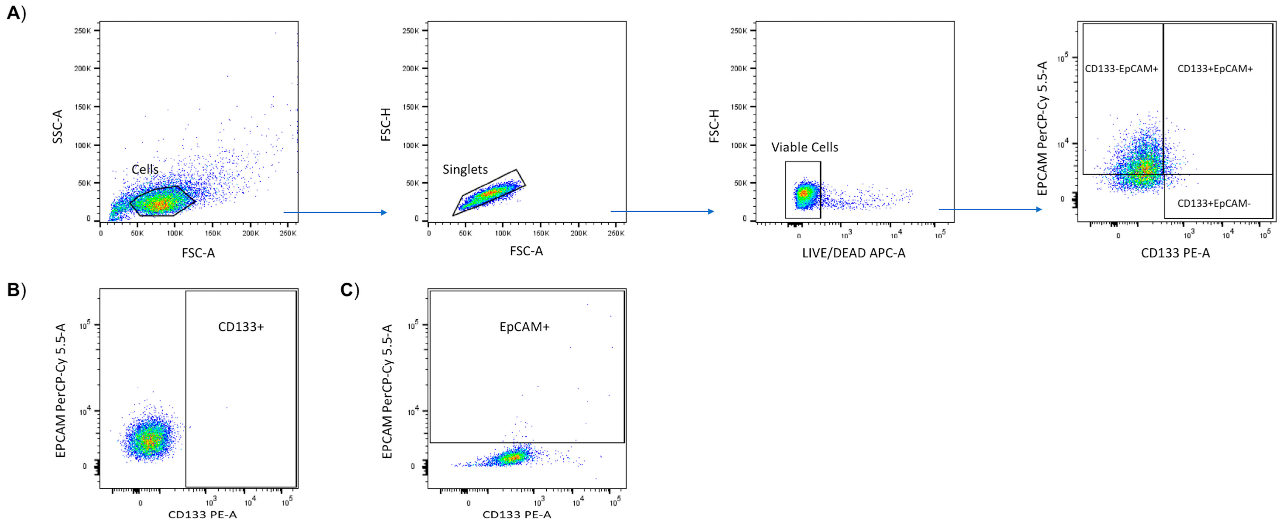

Figure S2. Chromatographic analyses of DF5 for evaluating its stability in assay conditions (72-hour incubation; RPMI 1640 medium enriched with 10% of Fetal Bovine Serum, FBS). HPLC chromatograms are referred to only RPMI 1640 medium, 10% FBS (**A**); **DF5** (250 µM) at time = 0 (**B**); **DF5** (250 µM) at time = 72 hours (**C**).

A)

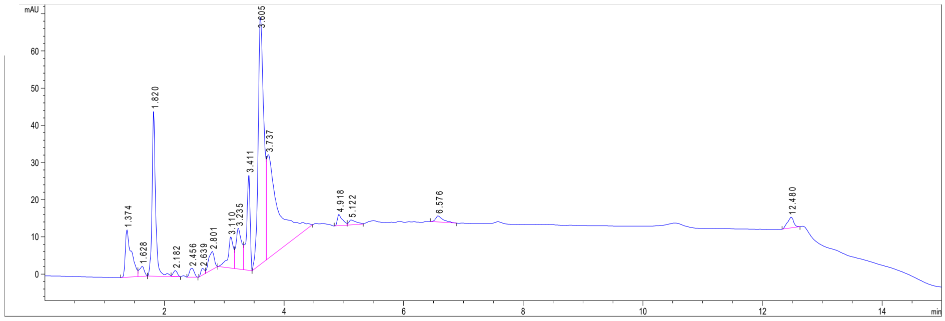

B)

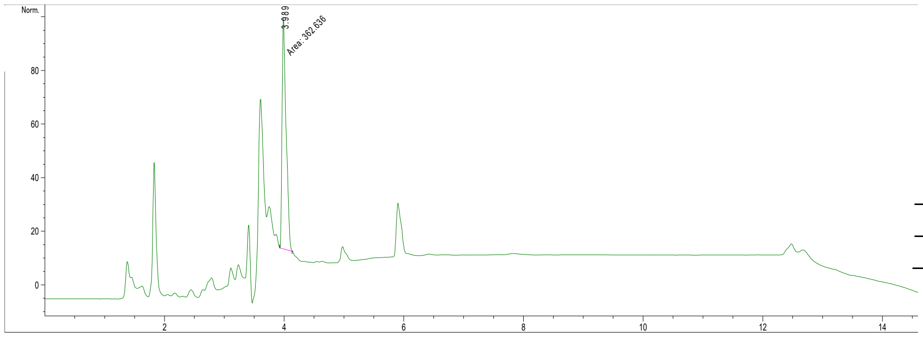

**Retention Time (min)**	**Area**
3.99	362.636

C)

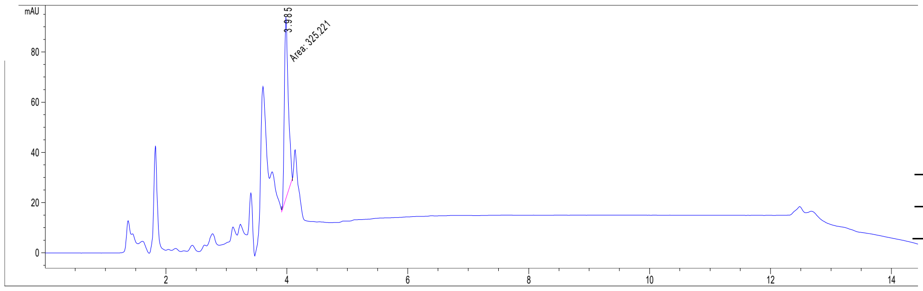

**Retention Time (min)**	**Area**
3.99	325.221

Table S1. Degradation percentage of DF5 over a 72-hour incubation.

**Time (h)**	**DF5 %**
0	100
72	89.68

Chromatographic conditions

The chromatographic analyses were accomplished through an Agilent 1260 Infinity II HPLC (Agilent, Santa Clara, CA, USA) consisting of a 1260 Infinity II Quaternary Pump (model G7111A), 1260 Infinity II auto-sampler (model G7129A), a 1260 Infinity II Multicolumn Thermostat (model G7116A), and a 1260 Infinity II Diode Array Detector (model G7115A) equipped with a Poroshell 120 EC-C18 column (150 x 4.6 mm i.d., particle size 4 μm, Agilent, Santa Clara, USA) operating at 20 °C.

The analyses were performed using as a mobile phase, a mixture of water (**A**) and acetonitrile (**B**) enriched with trifluoroacetic acid (0.1% *v/v*) in a gradient elution mode starting from 90% to 10% of water over 15 min (**Table S2**) at a flow rate of 1 mL/min. **DF5** was dissolved in DMSO and diluted in RPMI 1640 medium enriched with 10% of FBS to give a final concentration of 250 μM. The sample was left under stirring at room temperature for 72 hours. At fixed time points, aliquots were collected and analyzed by HPLC, setting the UV detector at 254 nm.

Table S2. Chromatographic conditions.

Time (min)	A (%)	B (%)
0	90	10
3	10	90
10	10	90
12	90	10
15	90	10
